# Inputs of humic fluorescent dissolved organic matter via submarine groundwater discharge to coastal waters off a volcanic island (Jeju, Korea)

**DOI:** 10.1038/s41598-017-08518-5

**Published:** 2017-08-11

**Authors:** Jeonghyun Kim, Guebuem Kim

**Affiliations:** School of Earth and Environmental Sciences, Seoul National University, 1 Gwanak-ro, Gwanak-gu, Seoul, 08826 Korea

## Abstract

The abundance of fluorescent dissolved organic matter (FDOM) in the surface ocean plays a critical role in the growth of marine microorganisms and corals by affecting the optical properties (i.e., the penetration of UV radiation) of seawater. In general, it is known that rivers are the main source of FDOM to surface ocean waters. Here, however, we show that the concentrations of FDOM in coastal seawater off a volcanic island, Jeju, Korea, are dependent primarily on submarine groundwater discharge (SGD). Based on a significant correlation between ^222^Rn and salinity in seawater, fresh groundwater was found to be the main source of groundwater as well as fresh water in the bay. The addition of dissolved organic carbon (DOC) and protein-like FDOM to the bay via SGD was generally negligible or negative. However, SGD enhanced the inventory of humic-like FDOM (FDOM_H_) in seawater by 2–3 times over all seasons, with conservative behavior of FDOM_H_ in bay seawater. These results suggest that SGD-driven fluxes of FDOM regulate its inventory in seawater and consequently play a significant role in determining the optical properties of coastal waters off islands and associated coastal ecosystems (i.e., corals).

## Introduction

Fluorescent dissolved organic matter (FDOM) is a major fraction of the sunlight absorbing dissolved organic matter (DOM) in water^1^. In particular, it absorbs harmful UV and visible radiation with a short wavelength^2^. Thus, the abundance of FDOM in the surface water influences optical conditions in aquatic environments^3^. It has been reported that FDOM contributes significantly to coastal ecosystems in terms of optically dependent components, such as the growth of coral reefs, juvenile fish, and photosynthesis^4–8^. FDOM is divided mainly into two different components, i.e., humic-like FDOM (FDOM_H_) and protein-like FDOM (FDOM_P_), depending on its origin and optical properties^9^. Typically, the fluorescence spectra of FDOM_H_ show broad and long excitation and emission wavelengths, and its peak location is similar to that of humic and fulvic acids^9^. The fluorescence spectra of FDOM_P_ show relatively narrow and short wavelengths, and its peak location is similar to that of amino acids (e.g., tryptophan and tyrosine amino acids)^10^.

In nearshore coastal oceans, both FDOM components are produced by autochthonous and allochthonous processes in waters and sediments^9^. Active photosynthesis and subsequent biological processes result in the production of FDOM_P_ in the euphotic zone. On the other hand, microbial degradation of organic debris produces FDOM_H_ in the water column and sediments^11, 12^. In addition, significant amounts of terrestrial FDOM are transported to oceans via rivers. River waters contain dissolved organic substances derived from the efflux from soils and produced *in situ* in the water. Thus, the distribution of FDOM shows significant relationships with salinity or other terrestrial tracers (e.g., lignin) in coastal oceans^13–15^.

More recently, in addition to riverine fluxes, submarine groundwater discharge (SGD) has been recognized as a potential source of FDOM in coastal oceans^16–20^. SGD is the submarine groundwater outflow across the interface between the ocean and the land, regardless of its salinities and chemical properties^21, 22^. Chen, *et al*.^16^ successfully used FDOM to identify the groundwater-driven DOM in the Florida Coastal Everglades. Nelson, *et al*.^18^ used the spectral characteristics of FDOM as a tracer for identifying two different SGD sources in the coral reefs of Hawaii. In other studies, it was reported that, in subterranean estuaries (STE), the concentrations of FDOM in groundwater were considerably higher than those in seawater^17, 19^. In STE, FDOM showed either non-conservative behavior in the Florida coastal region^19^ and tidal flats^23^ or conservative behavior in waters off Jeju, a volcanic island in Korea^17^. However, a quantitative evaluation of SGD-driven FDOM in coastal waters has not been documented yet.

**Figure 1:**
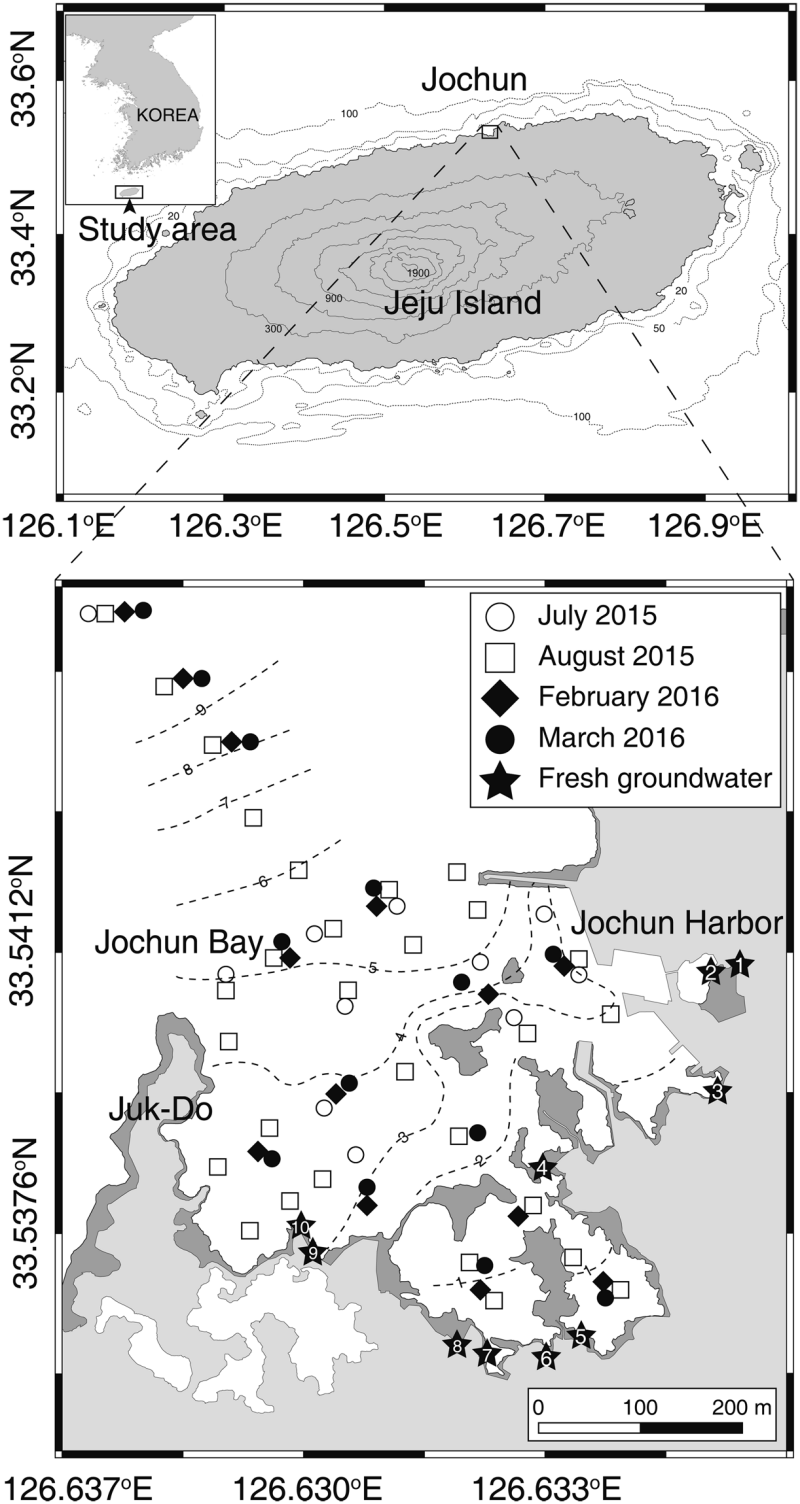
A map showing the study region and the sampling locations for DOC and FDOM in Jochun Bay in Jeju Island, Korea. Contour lines indicate the bottom depth based on *in situ* CTD casting. The number in the asterisk indicates the sampling station of the coastal spring well for the fresh groundwater. The maps and the sampling stations were drawn using Adobe Illustrator CC software version 2015.0.1. (http://www.adobe.com/).

Jeju Island is an ideal place for examining the influence of SGD-driven chemical constituents in the coastal ocean because most of the precipitation permeates through the porous ground and moves to the coastal ocean through SGD rather than surface runoff from the island, on the pathway of a branch of the oligotrophic Kuroshio Current^17, 24–27^ (Fig. 1). In this study, we attempted to determine (1) SGD-driven fluxes and behaviors of FDOM in a bay where SGD is pronounced, (2) seasonal changes in the contribution of SGD to the FDOM budget in the bay, and (3) the contribution of SGD to the budgets of protein-like and humic-like components of FDOM in the bay.

## Results and Discussion

**Figure 2:**
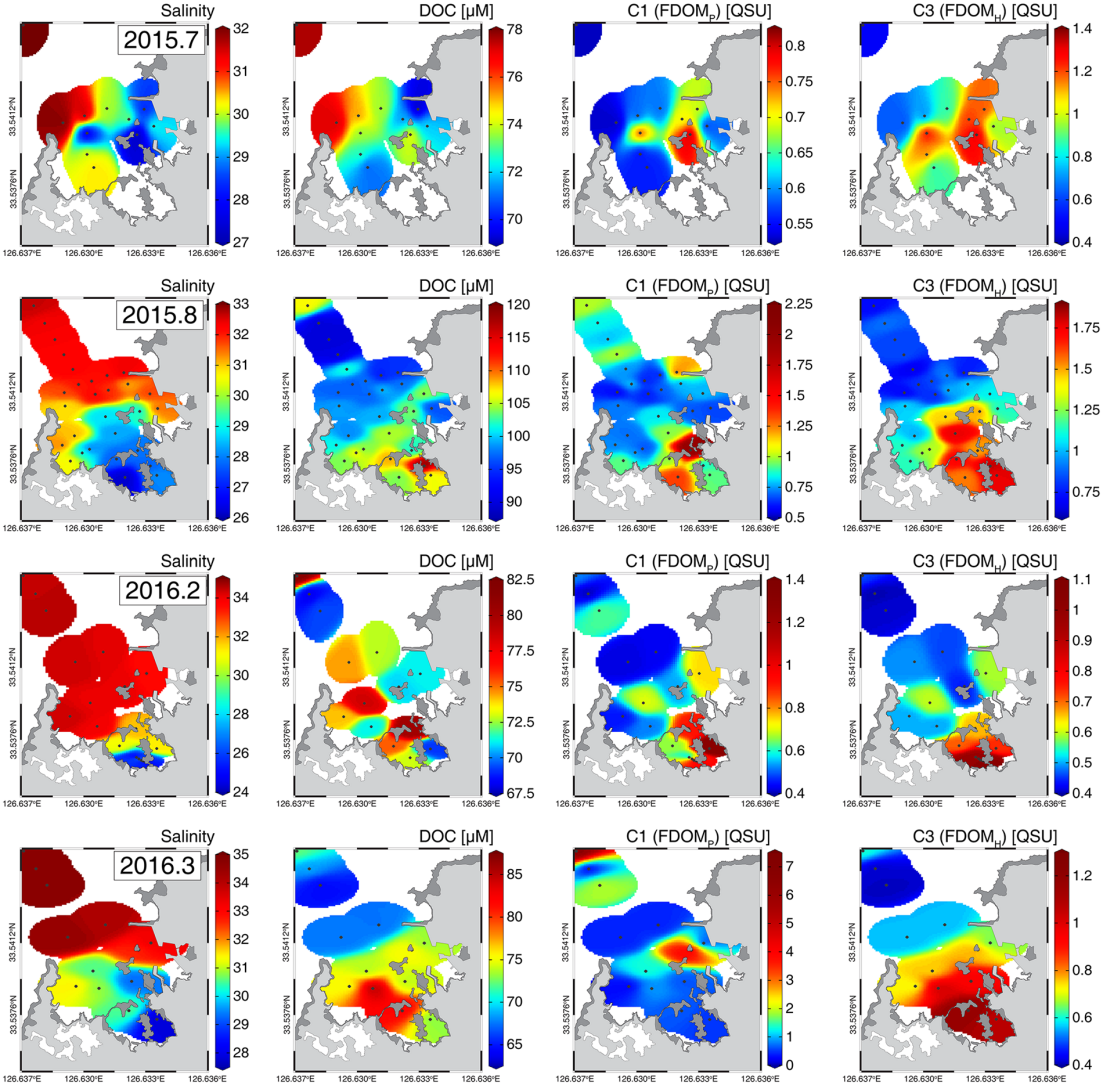
Distributions of salinity, DOC, FDOM_P_, and FDOM_H_ in the surface water in Jochun Bay. The contour plots were created using Ocean Data View software version 4.7.10. (https://odv.awi.de) and the background maps were drawn using Adobe Illustrator CC software version 2015.0.1. (http://www.adobe.com/).

The salinities of groundwater samples ranged from 0.17–6.87, with almost constant values (average: 1.24 ± 1.89) except for two samples in August 2015 (13.63 and 20.69) (Table S1). Based on a sharp density gradient in seawater, mainly due to the flow of fresh spring water, the surface layer (salinity: 31.06 ± 2.61) in the bay is separated from the deep layer (salinity: 33.51 ± 1.06) at approximately 1 m depth. Although the salinities of deep water in Jochun Bay were relatively constant during the four sampling campaigns, surface water salinities showed a large seasonal variation: low in summer (26.21–32.20, average: 29.96 ± 1.78, in July and August 2015) and high in winter (26.90–34.50, average: 32.05 ± 2.44, in February and March 2016). The lower salinity of seawater in summer seems to be associated with larger fluxes of fresh SGD due to heavy rainfall in the summer monsoon season. The contour plots of salinity in surface water showed lower salinity values in the innermost region of Jochun Bay (Fig. 2). These trends indicate that the main springs are located mainly in shallow depths because there are no rivers in the bay.

**Figure 3:**
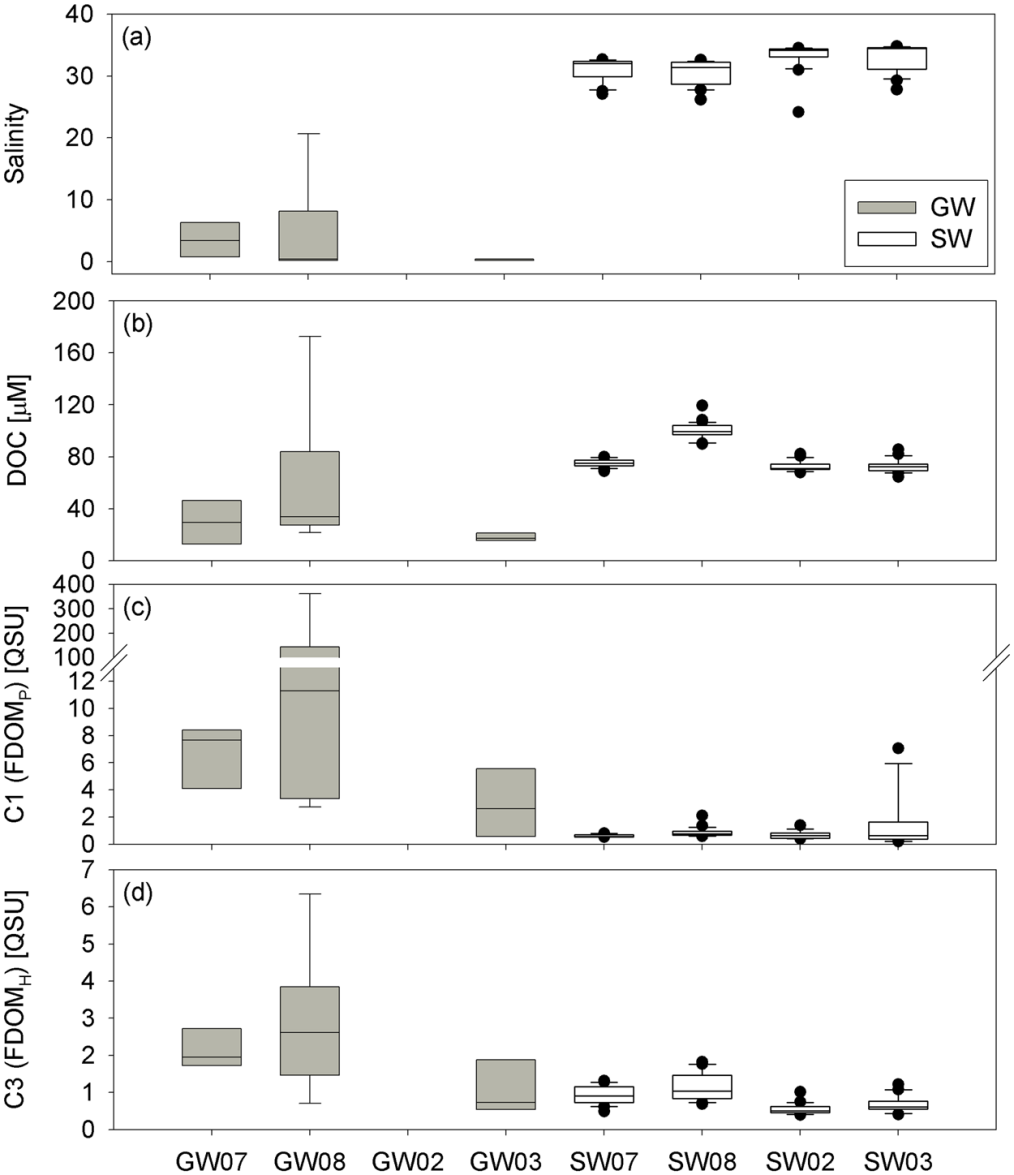
Box plots showing (**a**) salinity, (**b**) DOC, (**c**) FDOM_P_, and (**d**) FDOM_H_ in the groundwater (GW) and coastal seawater (SW) of Jochun Bay during four sampling campaigns.

**Figure 4:**
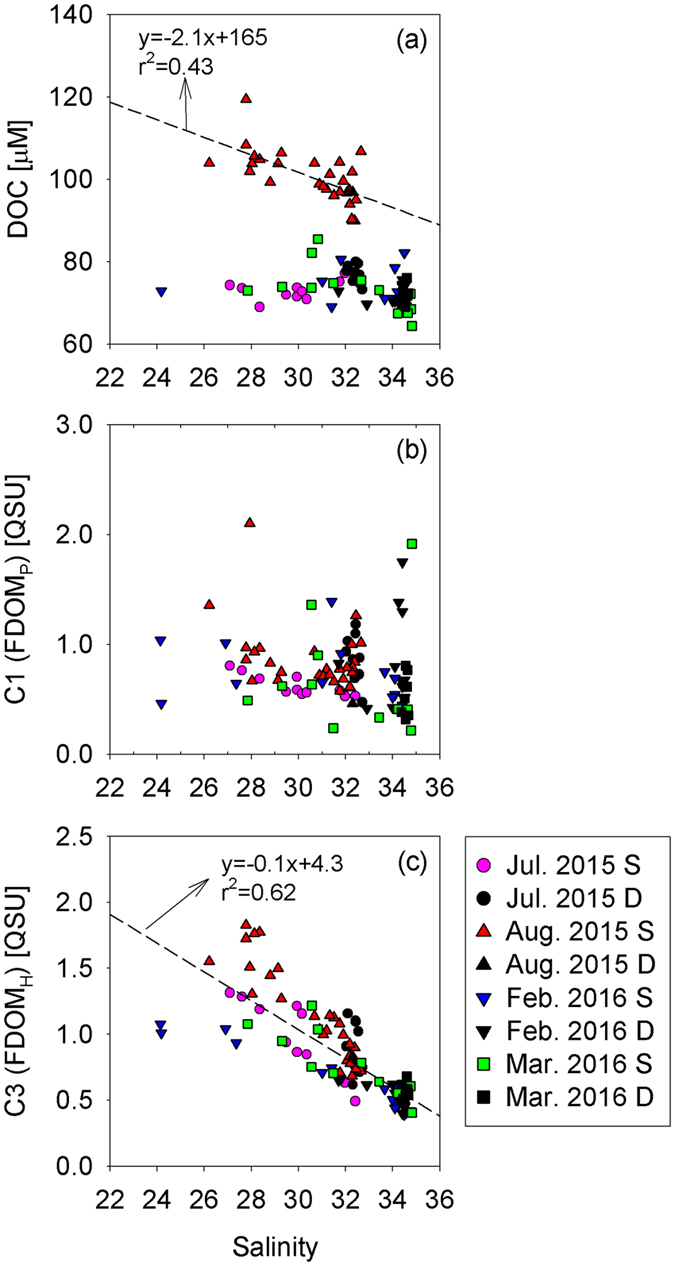
Scatterplots of the concentrations of (**a**) DOC, (**b**) FDOM_P_ and (**c**) FDOM_H_ against salinity in Jochun Bay. S and D represent the samples in surface and deep waters, respectively.

The average dissolved organic carbon (DOC) concentrations in the groundwater were 26 ± 11 μM during four sampling campaigns, except two unusually higher values (126 and 173 μM at wells 5 and 8, respectively) observed in August 2015 (Table S1). These DOC concentrations in groundwater samples were lower than those in seawater and rainwater samples (Fig. 3), typical of aged groundwater resulting from DOC degradation in the aquifer^17, 18^. In August 2015, relatively higher concentrations of DOC and a negative correlation between DOC concentrations and salinities (y-intercept: 165 μM) were observed (Table S2; Fig. 4). The y-intercept value was similar to the concentrations observed in the contaminated spring waters (i.e., wells 5 and 8). However, the y-intercept value was much higher than those in other wells (26 ± 11 μM). Thus, the high DOC concentrations in seawater in August 2015 seem to be linked to the introduction of unusually high, contaminated, DOC from hydraulically upgradient groundwater systems following heavy precipitation which occurred for three days (~90 mm/day) about one month before the sampling campaign. However, fluvial DOC inputs are unlikely since all sampling campaigns were performed at least one week after the precipitation event, which is longer than the water residence time of the bay.

The parallel factor analysis (PARAFAC) model identified three components from the 195 excitation-emission matrix spectroscopy (EEMs) data. The peaks of components 1 (C1) and 2 (C2) showed short excitation and emission spectra at Ex/Em = 300/360 nm and 320/336 nm, respectively. Based on a previous study, the location of the peak for C1 is similar to that of the protein-like “T” peak reported by Coble^9^. C2 is not exactly matched with the previous studies. However, C2 seems to be associated with FDOM_P_ because its concentration is highly correlated with that of C1 (r^2^ = 0.99). Component 3 (C3) showed broad excitation and emission spectra at Ex/Em = 325–365/400–450 nm. Its spectral location is similar to that of the terrestrial FDOM_H_ described as “C” peak in the previous study^9^. In this study, we use C1 and C3 to represent FDOM_P_ and FDOM_H_, respectively.

In general, all three components of FDOM in groundwater samples (average: 38.4 ± 89.9 QSU for C1, 25.2 ± 56.4 QSU for C2, and 2.1 ± 1.4 QSU for C3) were approximately 2–45 times higher than those in seawater samples (average: 0.9 ± 0.8 QSU for C1, 1.2 ± 1.4 QSU for C2, and 0.8 ± 0.3 QSU for C3) (Fig. 3). Although C3 showed a significant correlation (r^2^ = 0.62) against salinity in surface and deep seawater, C1 showed large scattering against salinity. This difference suggests that the sources and behaviors of FDOM_P_ and FDOM_H_ are different in this bay.

The relationships between salinity and C1 (FDOM_P_) in the surface water were relatively significant in July and August 2015, but not in February and March 2016 (Table S2; Fig. 4). The distributions of C1 in surface seawater and groundwater (Table S1; Fig. 4) suggest that the relative enrichment of FDOM_P_ in most of the groundwater influences the high FDOM_P_ stations in summer seawater. The distributions of FDOM_P_ in the winter season showed larger scattering. In general, the deep water showed large variations of FDOM_P_ for a constant salinity, with unusually higher concentrations in some stations (Fig. 4). Thus, the concentrations of FDOM_P_ in this bay seem to be affected by various sources such as groundwater inputs, biological production, and vertical water mixing.

The concentrations of C3 (FDOM_H_) in groundwater samples were considerably higher than those in seawater (Fig. 3). The FDOM_H_ in surface seawater was negatively correlated with salinity (Table S2; Fig. 4), indicating that FDOM_H_ originated mainly from the fresh groundwater. The linear relationship between FDOM_H_ and salinity suggests its conservative behavior during mixing between the groundwater and seawater. The relatively higher concentrations of FDOM_H_ in summer seawater seem to be associated with higher concentrations in most of the summer groundwater samples (Table S1; Fig. 4). The FDOM_H_ concentrations and salinities in deep-water samples fall within the trend of the data for the surface layer samples (as open-ocean water background concentrations), although slightly higher FDOM_H_ concentrations were observed in near-shore deep-water samples owing to surface-water mixing. The distribution patterns also clearly show that fresh groundwater is a dominant source of FDOM_H_ in this region (Fig. 4).

In order to determine the contribution of the groundwater-driven DOM in Jochun Bay, the flux of DOM from the coastal groundwater was compared with that from the open ocean during the water residence time of the bay. Based on the tidal prism model, the residence time of Jochun Bay was 1.0, 0.8, 0.6, and 1.3 d in July 2015, August 2015, February 2016, and March 2016, respectively^28^. The volume of the bay is 7.4 × 10^5^ m^3^, and its mean depth is 2.3 m. Based on the ^222^Rn mass balance model, the fluxes of submarine fresh groundwater discharge (SFGD) in Jochun Bay were estimated to be 6.9, 5.5, 5.0, and 4.1 (×10^4^) m^3^ d^−1^ in July 2015, August 2015, February 2016, and March 2016, respectively^28^. The volume of SGD accounted for approximately 4–9% of the total water volume in Jochun Bay during the water residence time. In Jeju Island, precipitation is concentrated in the summer monsoon season (June–September) and is strongly associated with the seepage rate of groundwater^24^. The relatively higher discharge rates of SFGD in July and August 2015 seem to be supported by the lower salinity of the surface water (average: 29.91 and 30.56), as compared to that in February and March 2016 (average: 31.72 and 32.29). The SFGD originating from the main coastal springs seems to affect the surface layer (within a depth of 1 m) based on the gradient of salinity.

Combining all data from four sampling campaigns, one endmember of FDOM_H_ in the fresh groundwater was derived from the y-intercept of the relationship between FDOM_H_ and salinity in the coastal seawater (Fig. 4). However, the y-intercept value was not in good agreement with the measured concentrations of FDOM_H_ in the spring waters. This may be due to the various hidden pathways of the aquifer and various contributions from each spring well. This result suggests that the averages of DOC and FDOM in groundwater can be decoupled from the actual endmember of groundwater owing to the complex nature of groundwater seepages. Therefore, the y-intercept value of the correlation between FDOM and salinity in the coastal seawater is a more reliable and representative endmember. Moreover, these results suggest that conservative organic substances (i.e., DOC and FDOM) can provide further information for locating SGD sources and pathways, in addition to natural SGD tracers^18^.

**Figure 5:**
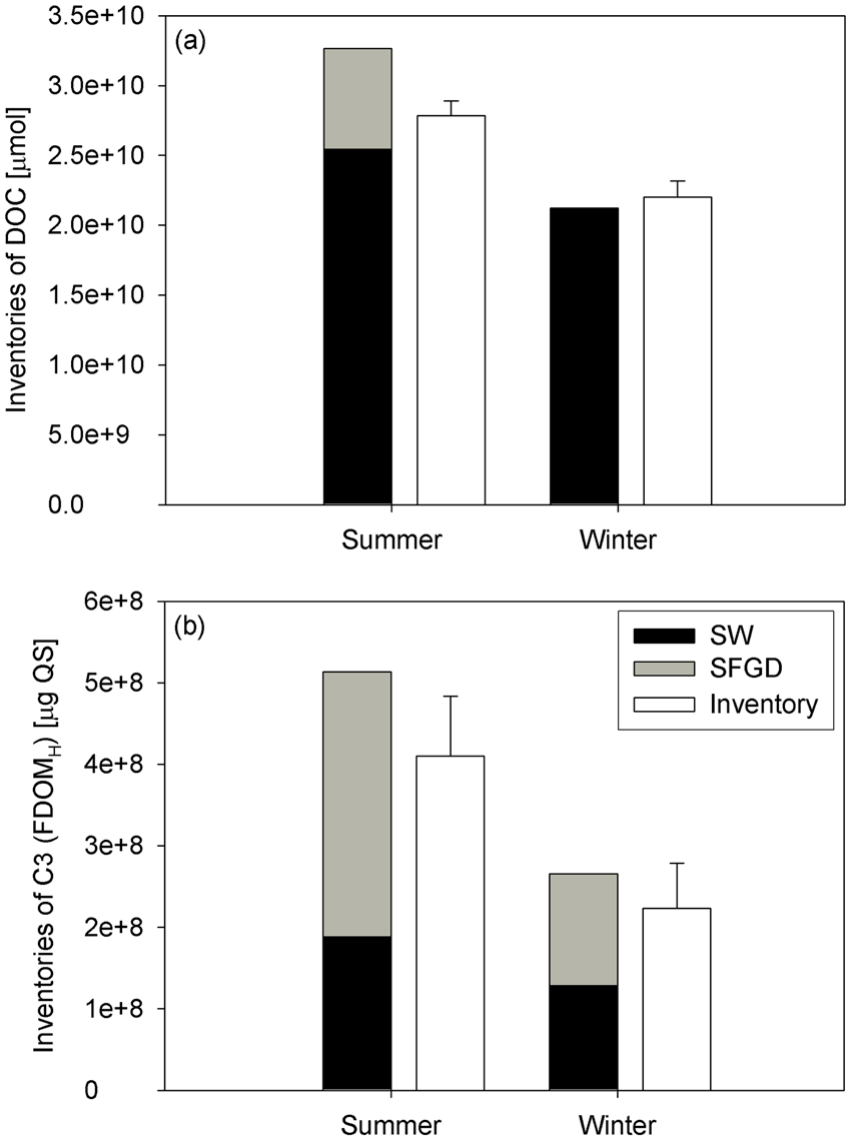
Inventories of (**a**) DOC and (**b**) FDOM_H_ derived from SFGD and seawater, and the actual inventories in Jochun Bay in summer (July and August 2015) and winter (February and March 2016).

Therefore, in this study, we calculated the inventories of DOC in summer and FDOM_H_ in all seasons, which showed a significant correlation against salinities. Here, the inventory is obtained by multiplying the fluxes of DOC and FDOM_H_ (μmol per day and μg QS per day) by the water residence time in the bay (day). During the water residence time in the bay, the inventories of FDOM_H_ originating from the fresh groundwater in summer were higher relative to those in winter (Fig. 5). The endmembers of FDOM_H_ in the fresh groundwater were 5.4 and 6.4 QSU in summer and 2.6 and 3.8 QSU in winter (Table S2). Using the endmember of FDOM_H_ and the rate of SFGD, the inventories of FDOM_H_ resulting from SGD were estimated to be 3.3 × 10^8^ and 1.4 × 10^8^ μg QS in summer and winter, respectively (Fig. 5). The background inventories of FDOM_H_ originating from the open ocean were calculated using the endmember value of FDOM_H_ in the open ocean water and the volume of the surface water layer. The endmembers of FDOM_H_ in the offshore were 0.49 and 0.68 QSU in summer and 0.40 and 0.41 QSU in winter. The background inventories of FDOM_H_ originating from the seawater were estimated to be 1.9 × 10^8^ and 1.3 × 10^8^ μg QS in summer and winter, respectively (Fig. 5). The measured inventories of FDOM_H_ in Jochun Bay were calculated by multiplying the average concentration of FDOM_H_ by the volume of the surface layer. The sum of the inventories of FDOM_H_ originating from fresh groundwater and the open ocean seawater during the water residence time was similar to the measured inventory in Jochun Bay (Fig. 5), indicating that the estimations are reasonable.

In the bay waters, the contributions of FDOM_H_ originating from the groundwater accounted for 170% and 110% of the offshore water in summer and winter, respectively (Fig. 5). In contrast, the contributions of DOC originating from SGD in the coastal seawater were negligible or lower than those from seawater in July 2015, February 2016, and March 2016 (Figs 3 and 4). The inventory of DOC originating from SFGD in the exceptional season (summer) was estimated to be approximately 7.3 × 10^9^ μmol (Fig. 5), which accounted for approximately 28% of the background offshore water. These results suggest that SGD is a significant source of FDOM_H_ but an insignificant source of DOC in coastal waters off volcanic islands. This seems to be associated with the production of FDOM_H_ in coastal aquifers due to microbial activity, resulting in the production of fulvic and humic acids, during DOM degradation. Therefore, SGD-borne FDOM_H_ is determined to be an important hidden source of FDOM_H_ in coastal waters, which is critical for coastal ecosystems including corals, seaweeds, and biological production. Corals need sunlight for building reefs by symbiotic zooxanthellae, but ironically they are highly vulnerable to harmful UV radiation. Zepp, *et al*.^6^ reported that FDOM accounts for 70–95% of the total UV attenuation in surface waters. Since FDOM efficiently protects the labile cells of microorganisms and corals from harmful UV radiation, FDOM is considered to be a key factor in sustaining the coral ecosystem^3, 4, 6, 29^. Additionally, high DOC concentrations are another major threat to coral reefs^30, 31^. The relatively low DOC concentrations in groundwater from the volcanic islands, Jeju^17^ and Hawaii^18^, can provide optimal conditions for the growth of corals. Thus, we surmise that SGD provides favorable conditions for the growth of coral reefs regarding the properties of organic matters in the ocean.

## Conclusions

In Jochun Bay, off the volcanic island, Jeju, SFGD accounted for 4–9% of the total water volume. The high enrichments of DOC, FDOM_P_, and FDOM_H_ in the bay seawater were linked to those in fresh groundwater inputs. In particular, based on the high concentrations of FDOM_H_ in the groundwater and its negative correlations with salinity in the seawater, SFGD accounted for approximately 170% and 110% of the background inventory originating from the open ocean water in summer and winter, respectively. However, the contribution of SFGD to DOC and FDOM_P_ inventory in seawater was negligible. This study highlights that SGD is a key factor controlling FDOM distributions in seawater, potentially affecting the optical and chemical conditions in coastal ecosystems. Because corals are susceptible to contamination by DOC and damage from strong UV radiation, the groundwater in Jeju Island potentially induces a favorable condition for the growth of coral reefs. More extensive studies are necessary to quantify the SGD-driven fluxes of FDOM in coastal seawater, particularly in islands where the optical properties significantly influence ecosystems.

## Materials and Methods

### Study region

Jeju Island is a volcanic island located in the southern sea of Korea, on the pathway of a branch of the oligotrophic Kuroshio Current (Fig. 1). Because the island bed is mainly composed of porous basalt rocks, most of the rainwater permeates through the porous ground and is transferred to the coastal ocean through groundwater^24^. Approximately 1000 artesian springs and wells are located along the coast. The groundwater from coastal springs and wells supports approximately 90% of the fresh water resources in Jeju Island. The study area, i.e., Jochun Bay, is located in the northern part of the island (Fig. 1). The bay is semi-enclosed by rocks and a harbor and has several coastal springs. The water residence time of the bay is approximately one day^28^. Owing to the East Asian monsoon system, heavy precipitation occurs in this region from June to September (~1900 mm per year).

### Sampling

Four sampling campaigns for seawater and coastal spring water were conducted in Jochun Bay, Jeju, Korea, in 2015 and 2016 (July and August 2015; February and March 2016) (Fig. 1). Seawater samples in the surface and deep-water layers were collected into acid-washed 1-L Nalgene Polypropylene bottles using a submersible pump. Within a few hours after the seawater sampling was completed, the fresh groundwater samples were collected from artesian spring waters into the acid-washed bottles by hand, using gloves (Fig. 1). Salinity was measured *in situ* using a conductivity-temperature-depth sensor (OCEAN SEVEN 304 CTD, IDRONAUT Co., Milano, Italy).

### FDOM analysis

The seawater and groundwater samples were filtered using pre-combusted Whatman GF/F filters with a pore size of 0.7 μm to remove large-sized materials. Filtered samples were transferred into pre-combusted vials and kept in a refrigerator at a temperature less than 4 °C until spectral analysis was performed. The filtrate was re-filtered using Nuclepore polycarbonate filters with a pore size of 0.2 μm to eliminate fine particles and microorganisms. Optical measurements of FDOM fluorescence were performed using a fluorescence spectrometer (FluoroMate FS-2, SCINCO, Korea) within one week after each sampling campaign. The scanning wavelengths of the excitation and emission spectra ranged from 250 to 600 nm (2 nm intervals) and 250 to 500 nm (5 nm intervals), respectively. The inner filter effect was not corrected for because the influence of this artifact was negligible (only 1–3% of the fluorescence intensity) for groundwater and seawater using this spectrofluorometer^23^. Rayleigh and Raman scatters were eliminated and interpolated based on Delaunay triangulation, which is developed by Zepp, *et al*.^32^, to prevent any problems with the blank subtraction method^32, 33^.

The PARAFAC model was applied to a combined set of 195 EEMs data. An appropriate number of components were evaluated statistically through the split-half test using the DOMFluor toolbox for MATLAB^34^. The concentrations of FDOM were normalized every day using the values of a quinine sulfate (QS) dihydrate diluted in 0.1 N sulfuric acid at a specific wavelength (Ex/Em = 350/450 nm). The concentrations are expressed in quinine sulfate equivalent units (QSU) (i.e., in μg QS/L). The variation of the measured QS fluorescence was less than 3% of the average value throughout the all measurements.

### DOC analysis

Samples for DOC were vacuum filtered simultaneously with FDOM samples using pre-combusted Whatman GF/F filters. To prevent microbial activity, the filtered samples were acidified using 6 M HCl, followed by hermetic sealing in pre-combusted (500 °C for at least 4 h) 20 mL glass ampoules (Wheaton Scientific, Millville, NJ). The concentration of DOC was measured using a TOC analyzer (TOC-V_CPH_, Shimadzu, Japan). The accuracy of DOC concentration was verified every day using deep sea reference (DSR: 41–44 μM for DOC, University of Miami) samples.

### Data Availability

The datasets analysed during the current study are available from the corresponding author upon reasonable request.

## Electronic supplementary material

Supplementary Information
