# Supplementary material for: Inputs of humic fluorescent dissolved organic matter via submarine groundwater discharge to coastal waters off a volcanic island (Jeju, Korea)
Source: Sci Rep. 2017 Aug 11;7:7921. doi: 10.1038/s41598-017-08518-5 (PMC5554202; doi:10.1038/s41598-017-08518-5)
Supplement: Supplementary file 1 — Supplementary Information [file 41598_2017_8518_MOESM1_ESM.doc]

*Scientific Reports*

Supplementary Information for

**Inputs of humic fluorescent dissolved organic matter via submarine groundwater discharge in coastal waters off a volcanic island (Jeju, Korea)**

Jeonghyun Kim and Guebuem Kim

School of Earth and Environmental Sciences, Seoul National University, 1 Gwanak-ro, Gwanak-gu, Seoul 08826, Korea

**Table S1. Salinities and concentrations of DOC, FDOM_P_ (C1), and FDOM_H_ (C3) in groundwater samples during three sampling campaigns**

|  | Station | Salinity | DOC  [μM] | C1  [QSU] | C3  [QSU] |
| --- | --- | --- | --- | --- | --- |
| *July 2015* | |  |  |  |  |
|  | 1 | 6.87 | 49 | 7.9 | 2.9 |
|  | 4 | 2.17 | 22 | 7.4 | 1.7 |
|  | 6 | 4.57 | 37 | 8.6 | 2.1 |
|  | 8 | 0.28 | 10 | 3.0 | 1.8 |
| *August 2015* | |  |  |  |  |
|  | 1 | 1.58 | 37 | 165.3 | 1.9 |
|  | 2 | 0.19 | 30 | 11.3 | 2.7 |
|  | 3 | 0.20 | 42 | 3.8 | 3.6 |
|  | 4 | 2.61 | 34 | 362.6 | 0.7 |
|  | 5 | 0.18 | 126 | 118.7 | 4.1 |
|  | 6 | 0.24 | 21 | 5.1 | 1.6 |
|  | 8 | 13.63 | 173 | 3.0 | 6.3 |
|  | 9 | 0.38 | 31 | 2.7 | 2.6 |
|  | 10 | 20.69 | 25 | 12.1 | 1.4 |
| *March 2016* | |  |  |  |  |
|  | 1 | 0.18 | 15 | 0.5 | 0.7 |
|  | 3 | 0.18 | 24 | 4.6 | 2.3 |
|  | 4 | 0.92 | 15 | 8.1 | 1.7 |
|  | 5 | 0.21 | 17 | 4.7 | 0.4 |
|  | 7 | 0.22 | 20 | 0.6 | 0.8 |
|  | 8 | 0.17 | 17 | 0.6 | 0.6 |

**Table S2.** The values of slope (s), y-intercept (y), and correlation coefficient (r^2^) for the correlations between salinities and the concentrations of DOC, FDOM_P_ (C1), and FDOM_H_ (C3) in surface waters in Jochun Bay

|  | s | y | r^2^ |
| --- | --- | --- | --- |
| *July 2015* (n=11) | | | |
| DOC | - | - | ns |
| C1 | -0.05 | 2.1 | 0.75** |
| C3 | -0.15 | 5.4 | 0.81** |
| *August 2015* (n=29) | | | |
| DOC | -2.13 | 165 | 0.43** |
| C1 | -0.07 | 2.8 | 0.17* |
| C3 | -0.17 | 6.4 | 0.84** |
| *February 2016* (n=17) | | | |
| DOC | - | - | ns |
| C1 | - | - | ns |
| C3 | -0.06 | 2.6 | 0.94** |
| *March 2016* (n=13) | | | |
| DOC | -1.45 | 120 | 0.35* |
| C1 | - | - | ns |
| C3 | -0.09 | 3.8 | 0.74** |

** p-value < 0.01

* p-value < 0.05

ns : not significant
